# A Comparative View of the Principal Theories, Which Have Prevailed in Chemistry, &c.

**Published:** 1799-05

**Authors:** 

**Affiliations:** Sen. of Vienna


### *A Comparative View of the principal Theories, which have prevailed in Chemiſtry, &c. By Dr.* FRANK, *Sen. of Vienna.*

[*Continued from Numb.* II. *pp.* 170—179.]

ACCORDING to the ideas of Dr. RICHTER, *light* is compounded of caloric, and that baſis which is an eſſential conſtituent part of all combuſtible bodies; the latter of theſe conſtituents, the author terms *brennſtoff*, or the *baſis of inflammability.*

The matters of *heat* and *light*, are, in his opinion, completely imponderable, and obey no laws of coercion. With reſpect to the former, he admits the principles eſtabliſhed by the antiphlogiſtic ſyſtem, in their full extent.

*Light* is generated only in the decompoſition of combuſtible bodies. The baſis of it is diſengaged from theſe, diſſolved or evolved in the matter of heat, and thus light is formed. This phenomenon, however, can take place only under the condition, that a *certain* and *determinate quantity* prevail in both elements. If this quantitive proportion does not ſubſiſt, for inſtance, if there be too little caloric, no light will ariſe, but merely heat.

Light paſſes through the pores of *tranſparent* bodies, without undergoing any change; but it is *decompoſed* by the *opaque.* They abſorb its baſis, and diſengage the caloric, with which the latter is combined. Colours and their different ſhades depend upon the degree of this decompoſition, or in other words, on the variouſly-modified proportion ſubſiſting between caloric and *brennſtoff.*

*Vital air* conſiſts of a *peculiar ſubſtance*, rendered elaſtic by means of caloric. Beſides this elaſtic form, vital air, as well as the baſis of light, and *brennſtoff*, can be obtained only by uniting with other bodies, which the author terms *ſubſtrata.* In combination with *nitrogen gas* (azote) it forms atmoſpheric air. The nitrogen gas is the baſe or matter of *nitre*, rendered rendered elaſtic by caloric, which matter again conſiſts of a *peculiar ſub-ſtratum*, and the *baſis of light*.

The *hydrogen gas* likewiſe conſiſts of a ſubſtratum combined with *brennſtoff* and *caloric*.

*Water* is compounded of the *baſe* of *vital air* and the *ſubſtratum of hydrogen;* it is produced when both ſubſtances are together ſubjected to inflammation in a gaſeous ſtate. The *brennſtoff* of the hydrogen gas then enters into combination with the caloric of the vital air, and forms light; thus the ſubſtratum of the former, and the baſis of the latter, become liberated, and unite in forming water.

A ſimilar double elective attraction takes place in every proceſs of combuſtion. The *brennſtoff* of combuſtible bodies, always combines with the caloric of vital air (by which alone the decompoſition of theſe bodies is practicable) and produces free light; but the ſubſtrata of combuſtible matters enter into union with the baſis of vital air, a proceſs by which the *mineral acid waters,* as well as the *calciform metals* are produced, the weight of which uniformly correſponds with the weight of the bodies ſubjected to combuſtion, and that of the vital air thus decompoſed.

In this manner, from a combination of the *baſis of vital air,* with the *ſubſtratum* of *carbon, ſulphur,* and *phoſphorus,* ariſe the *carbonic, ſulphuric,* and *phoſphoric acids ;* as, on the contrary, by the *ſubſtrata* of *metals,* are generated the *metallic calces.*

Combined with the *baſis of nitra,* the *baſis of vital air* generates the *nitric acid:* with the *baſis of muria,* the nature of which has not yet been diſcovered, the *muriatic acid* is produced.

All ſubſtrata which by their combination with the baſis of vital air have been dephlogiſticated, or deprived of their inflammable baſe, again return to their priſtine ſtate; if, in an elevated temperature, a phlogiſticated ſubſtratum be brought in contact with them; that is, ſuch a ſubſtratum as has a greater affinity with the baſe of vital air, than with that of light. The ſubſtrata exchange the baſe combined with each, and aſſume new forms. Such is the caſe in the regeneration of ſulphur and phoſphorus from their acids, as well as of the metals from their oxyds containing vital air (ſemi-acids), by treating them with carbon. This ſubſtance imparts its inflammable baſis to the ſubſtrata, and at the ſame time unites with their baſes of vital air, whereby it is changed into *fixed air* (carbonic acid gas.)

From the ſame cauſe, we can account for the decompoſition of water. The carbon deprives it of its baſe of vital air, and imparts its baſe of light to to the ſubſtratum of hydrogen. Thus ariſe the carbonic acid and hydrogen, both which obtain their gaſeous form, by the caloric diſengaged in this proceſs.

Some metallic ſubſtrata, however, combined with the baſe of vital air, are capable of decompoſing light, in an elevated temperature; of fixing its baſe, and thus of reaſſuming the metallic form: of this nature, for inſtance, are the oxyds of mercury; the baſe of vital air diſengaged on this occaſion, muſt neceſſarily appear in the form of gas, becauſe it is rendered elaſtic by the caloric there preſent *.

This conciſe outline of Dr. Richter's ſyſtem will be ſufficient to afford the reader a ſpecimen of his ingenious method of reaſoning. Some learned German chemiſts, indeed, have already paid homage to this new ſyſtem, among whom we obſerve the names of GREN † and LEONHARDI. This circumſtance, however, will not readily induce the adherents of the anti-phlogiſtic ſchool to adopt Richter's new ſyſtem of chemiſtry; nor is it probable, that the followers of Lavoiſier will be eaſily perſuaded to deſert the ſtandard of the French ſchool.

---

Among the rational ſceptics in chemiſtry, Profeſſor GOETTLING, of Jena, ſtands conſpicuous: he is equally diſſatisfied with the ſyſtem of Richter, and that of Lavoiſier, for explaining the phenomena of light. The obſervation, that phoſphorus emits light in azotic gas, and is changed into acid, induced him to make numerous experiments on this ſubject, from the reſults of which he draws the following concluſions, and thus attempts to ſettle the difference between the two ſyſtems.

The phenomenon of *light* depends on the liberation of a *peculiar element*, which he terms *lichtſtoff*, or the baſis of light.

This ſubſtance is a *conſtituent part* of all *inflammable* bodies.

Combined with *caloric*, which the author conceives according to the definition given of it by the antiphlogiſtic ſchool, but terms *baſis of fire*, he affirms that *lichtſtoff* forms *fire*.

*Oxygen* is not only evolved after being rendered elaſtic, by the influence of *caloric*, but likewiſe appears in a gaſeous form, combined with *lichtſtoff*. To the former combination (oxygen gas) he gives the name of *pyrogen* air; the latter (azote) he terms *lichtſtoffluft*, or *luminating air*. And from a mixture

---

* *On the Modern Objects of Chemistry*: By I. B. RICHTER. (in German) No. 3. 8vo. *Breslau and Hirschberg*. 1793.

† *Manual of Chemistry*: Second Edition, 4 vols, 8vo. Halle. 1794.

mixture of theſe two ſubſtances, he accounts for the compoſition of atmoſpheric air.

With *hydrogen*, (which, when combined with *lichtſtoff*, appears in the form of *hydrogenous gas) oxygen* produces *water*—an hypotheſis alſo conſiſtent with the principles of the antiphlogiſtic ſyſtem.

With the *baſes* of *carbon, ſulphur*, and *phoſphorus*, (which, in combination with *lichtſtoff* conſtitute *carbon, ſulphur*, and *phoſphorus)* Goettling endeavours to demonſtrate the compoſition of the *carbonic, ſulphuric*, and *phoſphoric acids.*

*Nitric acid* is, according to him, produced by an intimate combination of the *baſes of nitra* with the reſpective *matters of light* and *heat*.

The *muriatic acid*, as well as the *pure vegetable alkali*, apparently conſiſt of the ſame elementary baſes as the nitric acid, but united in *different* proportions.

The *vegetable acids* have a *ſaccharine matter* for their baſe, which acquires the nature of an acid, by being combined with *oxygen*.

*Metals* conſiſt of a peculiar metallic baſe and *lichtſtoff*. By their combination with oxygen, which they are capable of abſorbing in a greater or leſs proportion, they are changed into calces.

*Fixed alkalis* and *earths* are ſubſtances which *cannot be decompoſed*.

All combinations of *oxygen* and *lichtſtoff* take place, according to a double elective attraction, of which combuſtion affords an example. While the combuſtible body attracts the oxygen from the pyrogen air, its *lichtſtoff* combines with its pyrogen baſe, and appears in the form of fire. The combuſtible body itſelf acquires that proportion of weight, which is equal to the weight of the pyrogen gas that has eſcaped, and is changed either into water, acid, or metallic calx.

But it is equally poſſible that, during the ſaturation, mere light and no ſenſible heat ſhould be produced. Such is the caſe with phoſphorus, when placed in *lichtſtoffluft*. It deprives this luminating air of its oxygen, by reaſon of its greater affinity to the latter, and is changed into acid. Thus, the matter of light contained in both is diſengaged, and appears now in the ſtate of uncombined, or free light.

Bodies combined with oxygen are deprived of that ſubſtance, when they meet, in a higher temperature, with ſuch a *tertium* as manifeſts a greater elective attraction for oxygen, than for matter of light; as for inſtance, carbon. Its elementary baſe unites itſelf to the oxygen of the water, the acid, acid, and imparts its *lichtſtoff* to the elementary baſe of theſe combinations. They now return to their priſtine ſtate, but at the ſame time ſuffer that diminution of weight, which their oxygen before poſſeſſed *.

The facts on which Profeſſor Goettling endeavours to eſtabliſh his propoſitions, are unqueſtionably of great importance; and it is probable, that his theory, as it does not wound their national pride, will meet with a favourable reception among the German Chemiſts.

Time, the great improver of all diſcoveries; multiplied and careful reſearches; and unequivocal facts alone, can decide which of the ſyſtems we have now reviewed, is intitled to the greateſt ſhare of *probability*: for, in this inſtance, we cannot arrive at demonſtrative *certainty*.—Let it ſuffice, that we have here exhibited the outlines of thoſe ſyſtems. This, it will be readily conceived, has not been done with a view to improve the experienced chemiſt, but to inſtruct the phyſical ſtudent.

Dr. Frank concludes this part of the ſubject, by obſerving, that he was induced to compoſe the preſent treatiſe, in conſequence of the repeated wiſhes of his own pupils, as well as of a numerous branch of the profeſſors of pharmacy, many of whom are little acquainted with the laws and ſyſtems of chemiſtry; and who all complained of the want of a ſufficiently conciſe, and perſpicuous view of the different chemical ſyſtems.—" Nobody," ſays he, " can be more ſenſible, than I am myſelf, of its imperfections; but the general nature of the view I meant to give, induced me to confine it in this narrow compaſs, and rather to incur the blame of being too brief, than too diffuſe. A more particular account of the antiphlogiſtic ſyſtem would not have enabled the general reader to form ſuch an idea of it, as to underſtand and compare it with the ſyſtems I have yet to delineate."

---

* "Contributions to the improvement of the Antiphlogistic System of Chemistry;" By I. F. Goettling, 8vo. *Weimar*, 1764.

(To be concluded in the next Number.)

