# Errata

**Published:** 1799-07

**Authors:** 


					Xs'o. in. p. 273, in the note, tor "1764 read, 1794.

				

